# Development of Thermo-Responsive Polycaprolactone–Polydimethylsiloxane Shrinkable Nanofibre Mesh

**DOI:** 10.3390/nano10071427

**Published:** 2020-07-21

**Authors:** Chia-Hsuan Hsieh, Nur Adila Mohd Razali, Wei-Chih Lin, Zhi-Wei Yu, Dwita Istiqomah, Yohei Kotsuchibashi, Hsing-Hao Su

**Affiliations:** 1 Department of Mechanical and Electro-mechanical Engineering, National Sun Yat-sen University, Kaohsiung 80424, Taiwan; B053021005@student.nsysu.edu.tw (C.-H.H.); d063020007@student.nsysu.edu.tw (N.A.M.R.); m073020065@student.nsysu.edu.tw (Z.-W.Y.); m083020087@student.nsysu.edu.tw (D.I.); 2 Department of Materials and Life Science, Shizuoka Institute of Science and Technology, Shizuoka 437-8555, Japan; kotsuchibashi.yohei@sist.ac.jp; 3 Department of Otorhinolaryngology, Kaohsiung Veterans General Hospital, Kaohsiung 81362, Taiwan

**Keywords:** shape memory polymer, nanofibres, electrospinning, thermo-responsive

## Abstract

A thermally activated shape memory polymer based on the mixture of polycaprolactone (PCL) and polydimethylsiloxane (PDMS) was fabricated into the nanofibre mesh using the electrospinning process. The added percentages of the PDMS segment in the PCL-based polymer influenced the mechanical properties. Polycaprolactone serves as a switching segment to adjust the melting temperature of the shape memory electro-spun PCL–PDMS scaffolds to our body temperature at around 37 °C. Three electro-spun PCL–PDMS copolymer nanofibre samples, including PCL_6_–PDMS_4_, PCL_7_–PDMS_3_ and PCL_8_–PDMS_2_, were characterised to study the thermal and mechanical properties along with the shape memory responses. The results from the experiment showed that the PCL switching segment ratio determines the crystallinity of the copolymer nanofibres, where a higher PCL ratio results in a higher degree of crystallinity. In contrast, the results showed that the mechanical properties of the copolymer samples decreased with the PCL composition ratio. After five thermomechanical cycles, the fabricated copolymer nanofibres exhibited excellent shape memory properties with 98% shape fixity and above 100% recovery ratio. Moreover, biological experiments were applied to evaluate the biocompatibility of the fabricated PCL–PDMS nanofibre mesh. Owing to the thermally activated shape memory performance, the electro-spun PCL–PDMS fibrous mesh has a high potential for biomedical applications such as medical shrinkable tubing and wire.

## 1. Introduction

During a medical surgery, the knot-tying skill plays a vital role in the typical surgical suture, as the manipulation of the tying force depends on the medical operators. A tight suture treatment can lead to formation of a bean-like scar. Similarly, the dermis layer would be incompletely fixed which could result in overgrowth of fibroblasts in the wound gap and create scars. The research team developed a smart shape memory surgical suture which is made of polyurethane, and this biodegradable suture contains a tuneable shape property which can be controlled by changing the temperature [1]. Besides, recent research has also shown that the shape memory polymer- (SMPs) fabricated surgical sutures could provide a steady and uniform restoring force lasting more than two weeks for forming new tissue [2]. Temperature-responsive SMPs are smart materials that could be modulated to a temporary shape and can gradually recovered to their permanent shape upon exposure to heat [3,4]. Shape memory polymers have drawn great attention for their diverse biomedical applications such as smart sutures [1], drug deliveries [5] and cardiovascular stents [6].

Polycaprolactone (PCL)-based SMPs received much attention due to the fact of its various advantages such as biocompatibility, biodegradability and elasticity. Polycaprolactone (PCL) is useful as a switching segment for SMPs, as its melting temperature of PCL serves as the transition temperature which can be adjusted from 45 to 60 °C with increasing M_n_ [3], and this range is useful for deployment in vivo. The switching segment and soft segment are important criteria of SMP due to the fact of their dissimilarity in the structure and thermodynamic incompatibility. Besides PCL, PDMS is also widely used due to the fact of its remarkable thermal properties. Polydimethylsiloxane (PDMS) has an extremely low glass transition temperature (T_g_ = −125 °C) which makes PDMS an effective soft segment that can adjust the mechanical properties of PCL-based SMPs. It is mainly used for biomedical and food applications, as it are a non-toxic polymer. In general, PDMS has high performance, for example, in biocompatibility, low viscosity, good thermal properties, and chemical stability.

Shape memory polymer nanofibres have high potential for application as surgical sutures due to the face of their mechanical performance. As a result of its simple and robust performance, electrospinning has been a key choice to fabricate nanofibres that could possibly mimic the extracellular matrix (ECM) of the cells [7]. Extensive research has been done utilising the electrospinning technique to fabricate nanofibre from a wide variety of materials and polymers with different nanofibre structures and constructions. Mechanical properties of the SMP were affected by the different ratios of the switching segment and soft segment. For example, Zhang et al. [3] used PDMS with different molecular weights to control the chain length of the switching segment, and the results showed that the smaller molecular weight of PDMS could make better polymer with a higher shape recovery ratio and yield stress. Meanwhile, it is reported that the molecular weight of hard segment could form a stronger polymer with better shape recovery ratio [5,8].

In this study, we synthesized a series of temperature-responsive PCL–PDMS nanofibres with different mixture ratios of PCL and PDMS. The PCL–PDMS raw material was synthesized from the polycaprolactone diol (PCL diol) and polydimethylsiloxane diol (PDMS diol) and was then utilised to fabricate the PCL–PDMS nanofibres samples through the electrospinning process. The addition of PDMS segment can enhance the deformability (i.e., % strain at break) which can benefit the stretchability and flexibility of the SMPs. A series of experiments were conducted to characterise the thermal, mechanical and biocompatibility of the fabricated PCL–PDMS copolymer nanofibre.

## 2. Materials and Methods

This section describes the fabrication and characterisation of PCL–PDMS copolymer. In brief, the copolymers were synthesized and transformed into nanofibres structure via a conventional electrospinning process. Then, the fabricated copolymer nanofibres were assessed based on the chemical structure, surface morphology, thermal, mechanical and shape memory performance. The following subsection provides a detailed description of the procedures conducted for the assessment.

### 2.1. Synthesis of PCL–PDMS Copolymer

**Figure 1:**
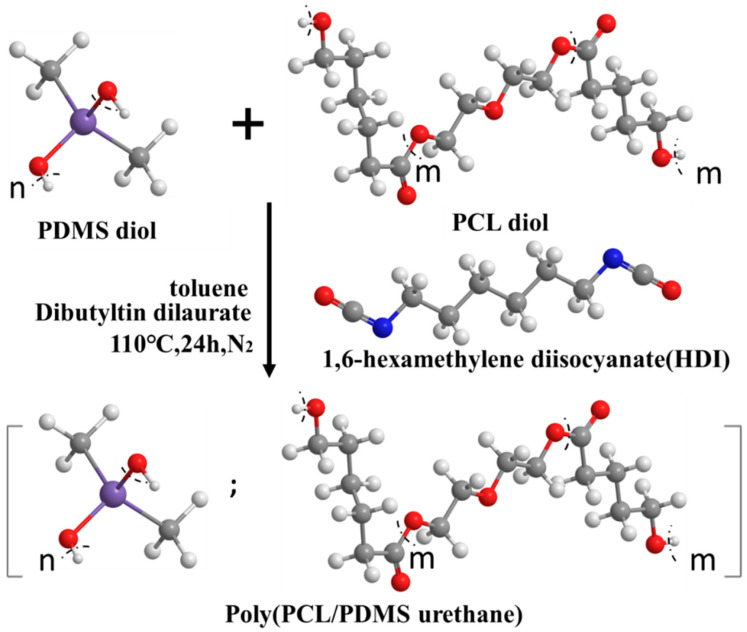
The synthetic procedure to fabricate the polycaprolactone-polydimethylsiloxane (PCL–PDMS) copolymer.

The PCL–PDMS copolymer was synthesized based on the protocol mentioned in [9,10]. The polyurethane synthesis was performed using polycaprolactone diol (PCL diol, M_n_ ~2000, Sigma–Aldrich, St. Louis, MO, USA) and dihydroxyl-terminated polydimethylsiloxane diol (PDMS diol, M_n_ ~4200, Sigma–Aldrich, St. Louis, MO, USA) at 6:4, 7:3 and 8:2 PCL–PDMS weight ratios. In this reaction, the PCL diol was regarded as a switching segment with a permanent shape property, while the PDMS diol was regarded as a soft segment with temporary shape property. In brief, different PCL and PDMS weight ratios were dissolved in 100 mL anhydrous toluene (99.8%, Sigma–Aldrich, St. Louis, MO, USA) at 60 °C in a round-bottomed flask, and 1.16 mL of hexamethylene diisocyanate (HDI, Alfa Aesar, Ward Hill, MA, USA) cross-linker was added equivalent to the reactive hydroxyl groups in the solution. Subsequently, 8 mg dibutyltin dilaurate (MSMD, ACROS Organics, Morris Plains, NJ, USA) catalyst was added into the flask. Then, the mixture was heated at 110 °C for 24 h under nitrogen atmosphere. N-hexane (99%, Sigma–Aldrich) was used as the solvent for precipitation of the resultant copolymer. Figure 1 shows the synthetic procedure of the PCL–PDMS block copolymer.

### 2.2. Fabrication of PCL–PDMS Copolymer Nanofibres

The PCL–PDMS copolymer solutions were prepared at 10 wt% with 1,1,1,3,3,3-hexafluoro-2-propanol (HFIP, 99%, Sigma–Aldrich) as solvent. The solutions were stirred overnight until homogeneous solutions were obtained. Then, the solutions were transferred to the electrospinning process to create electro-spun nanofibres. Each solution was transferred into a 3 mL syringe with a 24 gauge needle (diameter ~0.26 mm). The electrospinning process parameters were set at 15 kV applied voltage, flow rate at 0.225 mL/h, and the distance between needle and collector was kept at 19 cm. The solution was electro-spun on an aluminium foil and the fabricated nanofibres were dried in vacuum desiccator subjected to characterisation.

### 2.3. Characterisation of the PCL–PDMS Copolymer Nanofibres

#### 2.3.1. Nuclear Magnetic Resonance

To verify the chemical structure of the PCL–PDMS copolymer nanofibres, proton nuclear magnetic resonance, 1H NMR (JEOL ECZ600R, Tokyo, Japan) was utilised. The samples were prepared by mixing 3 mg of the copolymer nanofibres with 3 mL of chloroform-d (CDCL3, Sigma–Aldrich) solvent. The 1H NMR spectra were recorded at 400 MHz, at room temperature. Each sample was scanned 16 times to obtain the average spectrum. The chemical shifts corresponded relatively to the solvent at 7.3 ppm in 1H NMR spectra.

#### 2.3.2. Surface Morphology of the PCL–PDMS Copolymer Nanofibres

Scanning electron microscopy (SEM, JSM630, JEOL, Tokyo, Japan) was utilised to observe the fabricated nanofibres’ morphology including the size, shape and fibre diameter. Prior to SEM scanning, the samples were sputtered with gold and observed microscopically at an accelerating voltage of 10 kV. The nanofibres’ diameter was analysed manually from the SEM images using ImageJ software (version 1.44, National Institutes of Health, Bethesda, Maryland, USA). At least 200 diameters were randomly selected for each sample and the average nanofibre diameter was calculated.

#### 2.3.3. Thermal Properties of PCL–PDMS Copolymer

Differential scanning calorimetry (DSC, DSC-60 Plus, Shimadzu, Kyoto, Japan) was performed to assess the thermal properties of the copolymer including the degree of crystallinity and the melting temperature. The samples were sealed in perforated aluminium and the test temperature was set from 0 °C to 150 °C under a nitrogen (50 mL/min) environment at 10 °C/min heating rate. The melting point (T_m_) was determined by the peak of heat flow, and the crystallinity (*X_c_*) was calculated using the following equation:

(1) $$\%\;X_{c}=\frac{\Delta H_{m}}{\Delta H_{m}^{0}}\times 100$$

where Δ*H_m_* is normalised based on the % mass of PCL segments in the fibre 139.5 J/g for 100% crystalline PCL [11].

#### 2.3.4. Mechanical Properties of PCL–PDMS Copolymer Nanofibres

The ultimate tensile strength of the fabricated nanofibres was evaluated using a uniaxial tensile tester (FGP-0.5, Nidec-SHIMPO, Kyoto, Japan) at a rate of 20 mm/min at ambient temperature until failure. The samples were cut into a dimension of 30 mm × 5 mm × 100 µm (length × width × thickness) and clamped for uniaxial tensile test. Tensile strength, percentage elongation and Young’s modulus were determined from the linear region of the resultant stress–strain curve. The measurements were replicated five times.

#### 2.3.5. Shape Memory Behaviour

The shape memory behaviour of the copolymer samples were evaluated using strain-controlled cyclic thermomechanical tensile tests [3]. Rectangular samples with a dimension of 30 mm × 5 mm × 100 µm (length × width × thickness) were prepared and tested to the following processes sequentially. The shape memory effect was quantitatively measured as follows: (a) the samples were equilibrated at 40 °C for 10 min and stretched to 50% strain at a rate of 20 mm/min (*ε_m_* = 50%), (b) cooled to room temperature (25 °C) with an isothermal time of 3 min to fix the temporary shape, (c) unloaded to 0.01 N at a rate of 20 mm/min and measured the strain (*ε_f_*), and (d) re-heated the samples to 40 °C to recover the permanent shape and measured the strain (*ε_r_*). The processes were repeated for five cycles (*N*) for multiple cycle effects. The shape memory performances of the material were evaluated based on the ability of the material to maintain mechanical deformation after cooling for temporary deformation (shape fixity ratio, *R_f_*) and to return to its permanent shape after mechanical deformation (shape recovery ratio, *R_r_*) and as given [12]:

(2) $$R_{f}\left(N\right)=\frac{\varepsilon_{f}\left(N\right)}{\varepsilon_{m}}\times 100\%$$

(3) $$R_{r}\left(N\right)=\frac{\varepsilon_{m}-\varepsilon_{r}\left(N\right)}{\varepsilon_{m}-\varepsilon_{r}\left(N-1\right)}\times 100\%$$

where *ε_r_* (*N*) and *ε_r_* (*N* − 1) are the final strains in two successive cycle in the stress-free state during the recovery process, and *ε_f_* (*N*) is the ultimate strain in the stress-free state in the fixing process.

### 2.4. Cytotoxicity and Biocompatibility Study

Cell cytotoxicity and biocompatibility of the synthesized copolymer nanofibres were evaluated using primary dermal fibroblast (HDF) from American Type Culture Collection (ATCC). The HDF cells were cultured in Dulbecco’s modified Eagle’s medium (DMEM, Hyclone) supplemented with 10% foetal bovine serum (FBS, Hyclone) and 1% antibiotic–antimycotic (AAS, Hyclone) at 37 °C in 5% carbon dioxide (CO_2_) environment until it reached 80% confluency. Before seeding, the nanofibres were fixed in 24 well culture plates and sterilized under UV light for 2 h. The HDF cells were seeded at a density of 5 × 104 per each well, respectively. The culture plates were incubated at 37 °C with 5% CO_2_ and the culture medium was changed every other day.

The viability of the cells cultured on the nanofibres sample was monitored using Cell Counting Kit-8 (CCK-8, Sigma–Aldrich) assay according to the manufacturer instructions on day 1 and day 3. At each time point, 100 µL CCK-8 solutions in 1 mL culture medium was added into each well and further incubated for another 2 h. The absorbance was measured at 450 nm using a microplate reader (BIO-RAD Model 550, Tokyo, Japan). In addition, the biocompatibility of the material was observed through dual staining of fluorescein diacetate (FDA, Sigma–Aldrich, St. Louis, MO, USA)/DAPI, (4’,6-diamidino-2-phenylindole, Sigma–Aldrich, St. Louis, MO, USA). The HDF cells were cultured on the sterilized samples and at specific time intervals, the cultured cells were washed with PBS and stained with FDA (5 µg/mL, working concentration) and DAPI (0.1 µg/mL, working concentration) for 15 min to display live cells observed under a fluorescent microscope. Live cell morphology was observed with an inverted fluorescence microscope (Eclipse TS2, Nikon, Tokyo, Japan).

## 3. Results and Discussion

### 3.1. Molecular Characteristic of PCL–PDMS Copolymer Nanofibres by NMR

**Figure 2:**
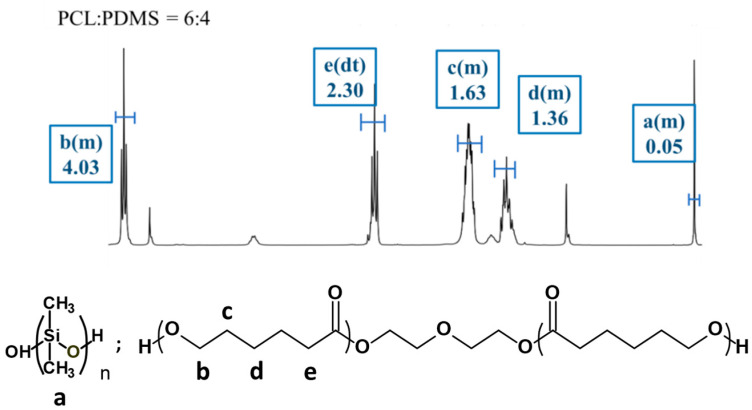
Compositional analysis of the PCL–PDMS copolymer nanofibres by ^1^H NMR spectroscopy.

The molecular characteristics of the fabricated PCL–PDMS copolymer nanofibres with different ratios were investigated using 1H NMR spectroscopy. Since all of the samples have similar peaks, only the spectrum for PCL_6_–PDMS_4_ is presented. Figure 2 shows the 1H NMR spectra for the PCL_6_–PDMS_4_ sample and the proton signals associated with both PCL and PDMS are observed. For the PCL segment, the peak detected at 1.63 ppm is attributed to the −(CH2)3−, 2.30 ppm for −CH2CO− and at 4.04 ppm for −OCH2− (methylene protons alpha to the ester group of PCL segments). Meanwhile, methylene protons in the repeated units of PDMS segments were observed at 0.05 ppm peak. The copolymer composition and the molecular weight of all copolymers were determined at 0.05 ppm (PDMS) and 4.04 ppm (PCL). Table 1 summarised the molecular properties of the PCL–PDMS copolymer nanofibres. Based on the calculated data, the weight ratio of the PCL and PDMS in all copolymer compositions was found to be consistent with the feed ratio, thus, confirming the successful fabrication of the PCL–PDMS copolymer nanofibres.

### 3.2. PCL–PDMS Copolymer Nanofibres’ Surface Morphology

**Figure 3:**
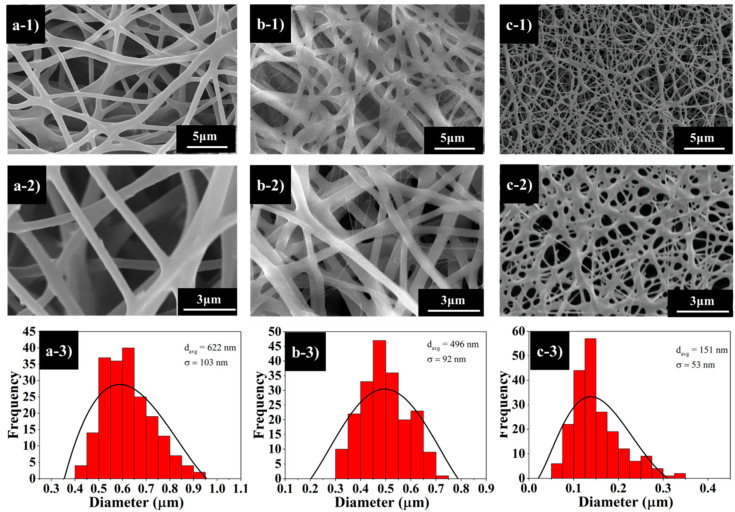
The scanning electron microscope (SEM) images of electro-spun PCL–PDMS copolymer nanofibres: (**a-1**,**a-2**,**a-3**) PCL_6_–PDMS_4_; (**b-1**,**b-2**,**b-3**) PCL_7_–PDMS_3_; (**c-1**,**c-2**,**c-3**) PCL_8_–PDMS_2_.

The PCL–PDMS copolymers were fabricated into nanofibre mesh via the conventional electrospinning process. The copolymer solutions were prepared at 10 wt% (w/w) diluted in HFIP and electro-spun at 10 kV. In general, with an appropriate polymer concentration, polymers with M_n_ > 10,000 will form continuous fibres during the electrospinning process [13]. Referring to Table 1, all the synthesized PCL–PDMS copolymer demonstrated M_n_ > 10,000 which is within the expected electrospinning fibre-forming range. Figure 3 illustrates the SEM images of the fabricated nanofibres from different copolymer composition ratios. The surface morphology of PCL–PDMS copolymer nanofibres show a continuous, smooth and bead-free structure with a random nanofibre orientation.

The PCL_6_–PDMS_4_ (Figure 3a) and PCL_7_–PDMS_3_ (Figure 3b) samples showed cylindrical and uniform nanofibres; meanwhile, the PCL_8_–PDMS_2_ sample showed a melted fibrous morphology. This might be related to the number-average molecular weight (M_n_) of the synthesized copolymer as obtained from the NMR spectroscopy (Table 1). The molecular weight of the synthesized copolymers decreased with the decreases of the PDMS mixture ratio. The melt fibrous morphology of the PCL_8_–PDMS_2_ sample indicated an insufficient physical cross-linking with less interconnected junction formed among the fibres. The average diameter of the copolymer nanofibres ranged from 151 nm to 622 nm when the PDMS composition increased from 20% to 40%. A larger nanofibre diameter was achieved with a sample with higher PDMS compositions. The increase in resultant nanofibre diameter was attributed to the molecular weight (M_n_) of the sample, where higher M_n_ will increase the fibre diameter due to the high number of chain entanglement and increased viscosity.

### 3.3. Thermal Properties of the PCL–PDMS Copolymers

**Figure 4:**
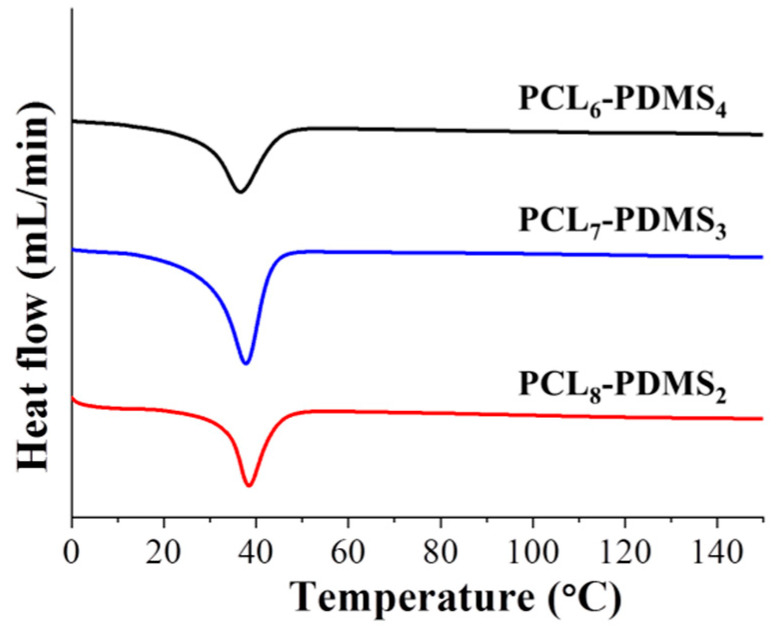
The differential scanning calorimetry (DSC) curves of the electro-spun PCL–PDMS copolymer nanofibres by the second heating run.

The thermal properties of the copolymer samples were characterised by DSC. The DSC curves after the second heating run were illustrated in Figure 4. The measurement was made after the second heating run to remove residual solvent and to erase the thermal history of the polymer during the synthesis process. It can be observed that the melting temperatures (T_m_) for all PCL–PDMS compositions were similar—approximately 37 °C. The T_m_ was used as the trigger temperature for shape recovery thus achieving a T_m_ close to the human body temperature which is very desirable for material intended to be used as biomedical material. The obtained result is consistent with a previous study, where the fabricated shape memory polymer showed a similar T_m_ around 38 °C [13].

Although the PCL–PDMS mixture ratio did not display any effect on the melting temperature, however, the crystallinity (X_c_) and enthalpy change (∆H_m_) showed the opposite effect. Both parameters demonstrated a positive correlation to the amount of PCL segment ratio. For example, the X_c_ increased from 21.93% to 26.83% when the PCL ratio increased from 60% (PCL_6_–PDMS_4_) to 80% (PCL_8_–PDMS_2_) of the copolymer ratio. Besides, the enthalpy change increased from 30.59, 34.82, and 37.42 (J/g) for PCL_6_–PDMS_4_, PCL_7_–PDMS_3_, and PCL_8_–PDMS_2_, respectively. The thermal properties of the fabricated PCL–PDMS copolymer nanofibres are presented in Table 2.

### 3.4. Mechanical Properties of the PCL–PDMS Copolymer Nanofibres

**Figure 5:**
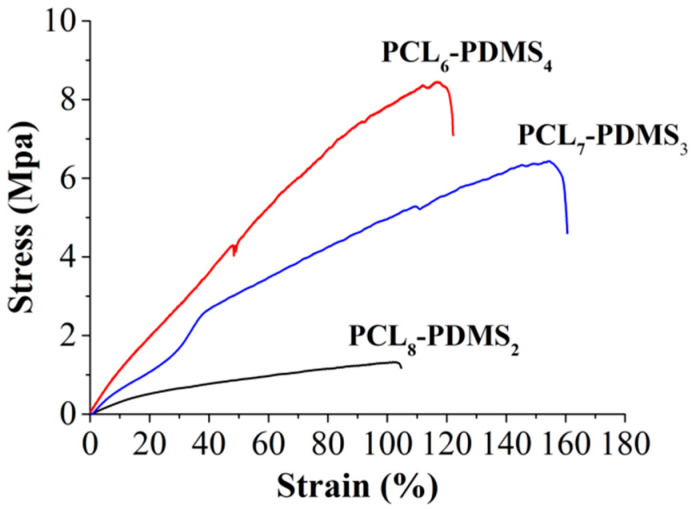
The stress–strain curves of the electro-spun PCL–PDMS copolymer nanofibres obtained from the tensile test.

Figure 5 illustrates the stress–strain curve of the copolymer nanofibres during the uniaxial tensile test. The tensile properties of the copolymer nanofibres such as tensile strength, percent of elongation and Young’s modulus are tabulated in Table 3. The tensile properties of the copolymer nanofibres were highly dependent on the copolymer mixture ratio. The tensile strength of the PCL_8_-PDMS_2_ is 1.41 MPa, and when the ratio of PDMS content increases to PCL_6_-PDMS_4_, the tensile strength increases to 6.64 MPa. In addition, the percent of elongation ranging between 122.66% to 154.44% and the modulus ranged from 3.42 MPa to 12.65 MPa with the addition of the PDMS soft segment. The obtained result differed from some published studies that stated that the higher PCL content increases the crystallinity of the copolymer fibres thus increase the tensile properties of the nanofibres [3]. This result might be attributed to the fabricated nanofibre morphology where the uniformity of the diameter of the PCL_6_–PDMS_4_ nanofibres could distribute the stress during the tensile test, therefore, providing a more stable structure. Meanwhile, the melted fibrous structure indicating insufficient physical cross-linking with less interconnected junction formed among the fibres [14] within the PCL**_8_**–PDMS**_2_** sample caused the reduction in tensile strength. Moreover, it was reported that the addition of the PDMS soft segment content improves the tensile properties of SMP due to the higher soft segment entanglement density [15]. The fact that the PDMS had low T_g_ (−125 °C) makes PDMS a particularly effective soft segment which should enhance the deformability [8].

### 3.5. The Shape Memory Behaviour

**Figure 6:**
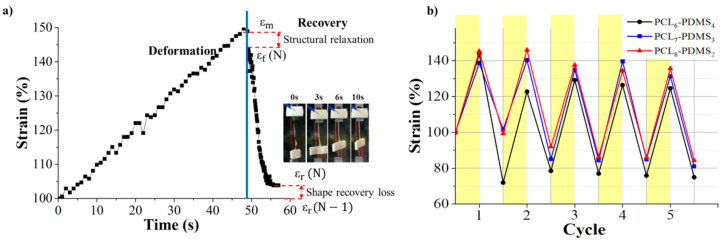
The shape memory behaviour of PCL–PDMS copolymer nanofibres: (**a**) one complete cycle as a function of time; (**b**) strain profile in five thermomechanical cycles.

The shape memory behaviour of the PCL–PDMS copolymer nanofibres was evaluated using thermomechanical cycles. The shape memory behaviour of the PCL–PDMS copolymer nanofibres for one complete cycle is shown in Figure 6a. The cycles involved programming the samples by heating at an elevated temperature, stretching to the desired shape at 40 °C (above T_m_) until the shape was fixed and cooling to a temperature lower than T_m_ (25 °C) so that the chain segment of the samples was in a temporary position before the stress was completely removed. The samples were then re-heated to 40 °C, relaxation occurred, and the temporarily deformed samples recovered to their original permanent shape. The time taken for complete shape recovery of the sample was also recorded. According to Figure 6a, it was observed that the recovery process started immediately after re-heating and achieved full recovery at approximately 10 s. Such a fast shape recovery of nanofibres in comparison to the film was achieved, due to the high surface area to volume ratio of nanofibres ensuring faster thermal conduction. [16]. The strain profiles of the five thermomechanical cycles are presented in Figure 6b. The shape memory performance in terms of shape fixity and recovery ratio for the PCL–PDMS copolymer nanofibres during thermomechanical cycles is tabulated in Table 4.

The PCL–PDMS copolymer nanofibres exhibited excellent shape fixity ratio which was above 83% for the first tensile cycle and exceeded 90% for subsequent cycles. The shape fixity of the copolymer nanofibres was lower in the first cycle because of the segment-chain orientation and relaxation effects [17,18]. As such, the shape memory nanofibres should be pre-conditioned to fully utilise the shape memory potential [13,17,18]. For subsequent cycles, the shape fixity of all samples surpassed 90%. After the fifth cycle, all samples maintained an excellent shape fixity performance up to 98% for PCL_6_–PDMS_4_. The samples were capable of maintaining the deformed shape and were not weakened regardless of the copolymer mixture ratio as proven by the excellent fixity ratio. The excellent fixity shown by the copolymer PCL–PDMS nanofibres was associated with the physical cross-links formed through inter- or intra-polymeric chain attractions such as hydrogen bonding or dipole–dipole interaction. Moreover, the degree of crystallinity for all samples obtained from the DSC was higher than 20% (Table 2) which allows for a high strain fixity ratio [19]. On the other hand, the shape recovery ratio for all samples was higher than 100%.

Our result shows the recovery ratio exceeded 100% which means that the length of the recovered SMP after heating was shorter than the original permanent shape. This might be due to the super-contraction behaviour of the nanofibre. The pre-orientation of the nanofibre during the electrospinning process leads to the super-contraction behaviour. At the temporary state, the nanofibres were pre-stretched causes a certain degree of molecular orientation and electrostatic repulsion. When the nanofibre was heated to recover the permanent shape, thermal expansion induced re-crystallization of the PCL. Rapid crystallization of PCL segments during the cooling process formed a more compact structure of the fibre [20]. The thermal expansion effect is neglected in the discussion. Besides, the shape recovery > 100% were reported in the previous reports for PCL–PDMS SMP [18,20]. The super-contraction behaviour of the SMP helps to maintain the shape after several cycles and prevent the fibre from sagging. In one report, the super-contraction behaviour of SMP helps to heal macroscopic damages as the fibres exert a higher compressive force on the damage interfaces due to the tendency to shrink further [16]. Since the thermomechanical cycles are repeatable, therefore, the capability to heal macroscopic damages may be repeatable. The PCL_6_–PDMS_4_ nanofibres with the largest diameter and amorphous domain yield the highest shape recovery in the first cycle (163.41%). Based on the result, it can be concluded that both the shape fixity and recovery ratio was independent of the number of cycles.

**Figure 7:**
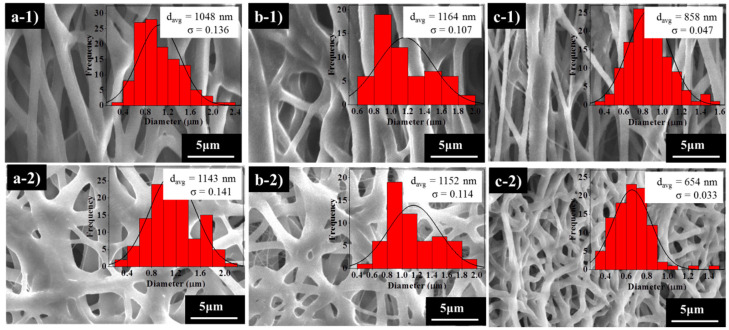
The SEM images of the nanofibres’ structure during the deformation and after recovery: (**a-1**,**a-2**) PCL_6_–PDMS_4_; (**b-1**,**b-2**) PCL_7_–PDMS_3_; (**c-1**,**c-2**) PCL_8_–PDMS_2_.

Furthermore, the nanofibres’ surface morphology during deformation and fixation after shape recovery was assessed, and the SEM images are presented in Figure 7. Different nanofibres structures are observed before and after the deformation. Initially, the fibres show a randomly oriented structure (Figure 3); however, after the temporary stretch (Figure 7a–c), the fibres tend to align along the strain direction. The samples maintained continuity and no broken fibres were detected, indicating the flexibility of the samples. Once the samples were re-heated, the directed nanofibres recovered to their original random orientation. The average nanofibres’ diameter increased in comparison to the initial state (Figure 3) after temporary deformation with the sample PCL_6_–PDMS_4_ showing the highest diameter increase from 348 nm to 1048 nm. Following the shape recovery, the average fibre diameter recovered to around 1143 nm, 1152 nm and 654 nm for PCL_6_–PDMS_4_, PCL_7_–PDMS_3_ and PCL_8_–PDMS_2_, respectively.

### 3.6. Cytotoxicity and Biocompatibility Study

**Figure 8:**
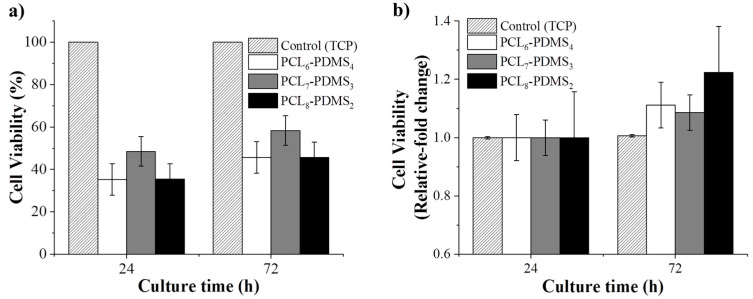
The biocompatibility of PCL-PDMS nanofiber mesh based on (**a**) viability percentage and (**b**) relative fold change relative to the control group.

The cytotoxicity of the fabricated nanofibres was statistically evaluated using the CCK-8 assay to validate their application in the biomedical field. Human dermal fibroblast cells were cultured on the fabricated nanofibres and the cytotoxicity effect was observed. The HDF cells cultured on a tissue culture plate (TCP) were regarded as the control group. Figure 8 shows the CCK-8 result for all samples after 24 h and 72 h. The viability is presented in terms of relative fold change from the control group.

The HDF cell viability is presented in terms of relative fold change from the control group. As expected, since the copolymer samples are based on derivatives of biocompatible PCL and PDMS, no samples showed any toxicity effect to the HDF cells and, as such, this demonstrates compatibility for biomedical application. According to Figure 8, the cellular viability for all samples increased significantly compared to the control group after 72 h, assuming that the nanofibres can promote cell growth and proliferation. This confirms that the fabricated copolymer nanofibre did not exhibit any toxicity to the cells for 72 h.

Although the fabricated nanofibres showed low cell viability percentage, this shrinkable nanofibre mesh can be applied as an early treatment on acute open wounded area during an emergency situation. Since the fabricated SMP was triggered at near body temperature (37 °C), the pre-stretched fibre mesh can recover to its initial shape upon applying to body. The shrinkage of the fibre during the shape recovery gives pressure to the wound site.

**Figure 9:**
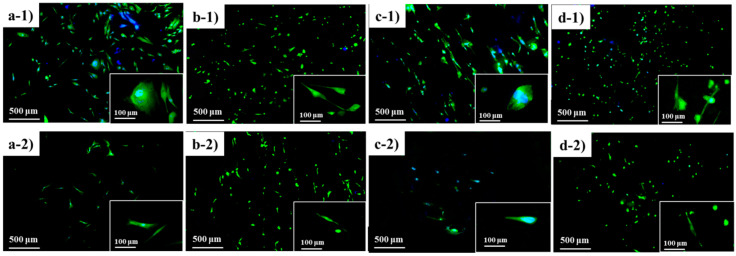
**Dual staining of fluorescein diacetate-4’,6-diamidino-2-phenylindole (**FDA/DAPI) for biocompatibility study: (**a-1**,**a-2**) control group (TCP); (**b-1**,**b-2**) PCL_6_–PDMS_4_; (**c-1**,**c-2**) PCL_7_–PDMS_3_; and (**d-1**,**d-2**) PCL_8_–PDMS_2_.

Besides, HDF cells seeded on the nanofibre samples were stained with FDA/DAPI double staining for observable evaluation on the cell morphology. The microscopy images of the cell morphology illustrated in Figure 9. Survival cells were observed in all samples with spreading cellular extensions.

### 3.7. Demonstration of the Shape Memory Response

**Figure 10:**
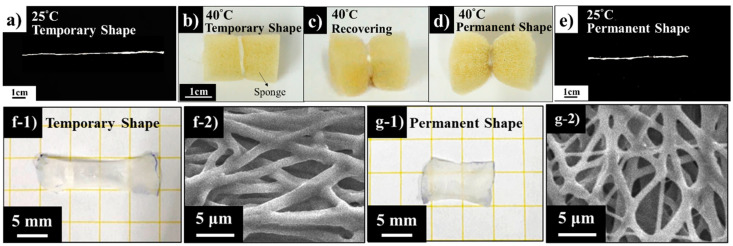
Visual demonstration of the nanofibres’ shape recovery performance: (**a,b**) temporary shape; (**c**) during recovery; (**d**) recovered to the permanent shape; (**e**) permanent shape; (**f-1**) temporary shape macroscopic view of nanofibre mesh; (**f-2**) temporary shape microscopic view of nanofibre mesh; (**g-1**) permanent shape macroscopic view of nanofibre mesh; (**g-2**) permanent shape microscopic view of nanofibre mesh.

The images in Figure 10 demonstrate the shape memory responses of the strip made up by electro-spun PCL_6_–PDMS_4_ nanofibres. To perform a visualized demonstration of shape memory responses, we wrapped a sponge with the PCL–PDMS strip which was programmed as a temporary shape state. Before that, the PCL–PDMS strip with 30 mm in length was, initially, stretched to 50% strain at 40 °C by using the tensile machine under the elongation speed of 20 mm/min. The strip was kept at 45 mm as a temporary shape state on the stage until the environmental temperature reached room temperature. As presented in Figure 10b, the ambient environment was heated again to 40 °C, and the stretched nanofibres shrunk to its initial length of 30 mm within 10 s as demonstrated in Figure 10d,e. Figure 10f,g showed the photos of nanofibre mesh which was cut into the size of 10 mm × 10 mm. Nanofibre mesh had undergone a series of temperature changes to demonstrate its shape memory ability. The nanofibre mesh was stretched to 200% at 40 °C (Figure 10(f-1)). After that, the nanofibre mesh was cooled to 25 °C until equilibrium was reached. Lastly, we could observe that after reheating nanofibre mesh to 40 °C, the nanofibre mesh recovered to the permanent shape (Figure 10(g-1)). As presented in the SEM image of Figure 10(f-2,g-2), we could conclude that the microstructure of nanofibre mesh changed with the shape of nanofibre mesh. The fibres showed the arranged orientation after being stretched (Figure 10(f-2)). Meanwhile, the energy generated by stretching was stored in the deformed nanofibre mesh. The increase in temperature caused the entropy change which, in turn, leads to changes in the macroscopic appearance. The fibre reverted to random orientation again (Figure 10(g-2)).

## 4. Conclusions

We fabricated and characterised polycaprolactone-based shape memory polymer (SMP) with different polydimethylsiloxane (PDMS) soft-segment mixture ratios. Depending on the PDMS soft-segment content, a series of PCL–PDMS copolymers, namely, PCL_6_–PDMS_4_, PCL_7_–PDSM_3_ and PCL_8_-PDMS_2_ fibres, were fabricated and engineered to nanofibre mesh structure with nanofibre diameter ranging from 151 nm to 622 nm. The fabricated PCL–PDMS copolymer can perform the shape changes with a large deformation triggered by temperature at approximately 37 °C. The mechanical properties of the PCL–PDMS copolymer fibres improve with the addition of PDMS soft segment. By comparing with the PCL_7_–PDSM_3_ and PCL_8_–PDMS_2_ fibre samples, PCL_6_–PDMS_4_ possessed the highest tensile properties with 6.64 MPa tensile strength and elongation of up to 122.7%. After five thermomechanical cycles, all PCL–PDMS fibre samples exhibited excellent shape recovery and shape fixity which was around 98% and 100%, respectively. The PCL–PDMS fibres demonstrated a fast response and required around 10 s to completely recover its original shape. Based on the experimental results, including appropriate mechanical properties, biocompatibility, along with excellent shape memory behaviour, we believe that the fabricated PCL–PDMS copolymer nanofibre mesh has great potential to be applied in biomedical application such as for shrinkable tubing and surgical suture.

## Figures and Tables

**Table 1:** Compositional analysis of the PCL–PDMS copolymer nanofibres by 1H NMR spectroscopy.

Sample	Feed Ratio (wt%)		Composition (wt%)		M_n_ (×10^3^)
	PCL	PDMS	PCL	PDMS	
PCL_6_–PDMS_4_	60	40	61.5	38.5	82.74
PCL_7_–PDMS_3_	70	30	69.6	30.4	37.00
PCL_8_–PDMS_2_	80	20	83.5	16.5	25.95

**Table 2:** The thermal properties of the PCL–PDMS copolymer samples measured by DSC.

Sample	X_C_ (%)	T_m_ (°C)	∆H_m_ (J/g)
PCL_6_–PDMS_4_	21.93 ± 2.1	36.44 ± 1.00	30.59 ± 2.9
PCL_7_–PDMS_3_	24.96 ± 1.9	37.41 ± 0.91	34.82 ± 2.75
PCL_8_–PDMS_2_	26.83 ± 2.7	37.70 ± 1.12	37.42 ± 3.8

T_m_ (melting temperature) and ΔH_m_ (enthalpy change) determined by the second heating run of DSC curves.

**Table 3:** The mechanical properties of electro-spun PCL–PDMS copolymer nanofibres.

Sample	Tensile Strength (MPa)	Elongation at Break (%)	Young’s Modulus (MPa)
PCL_6_–PDMS_4_	6.64 ± 1.5	122.66 ± 23.3	12.65 ± 3.9
PCL_7_–PDMS_3_	4.30 ± 1.5	154.44 ± 16.6	7.93 ± 1.0
PCL_8_–PDMS_2_	1.41 ± 0.57	137.52 ± 24.68	3.42 ± 0.79

**Table 4:** Shape recovery ratio (R_r_) and shape fixity ratio (R_f_) of the PCL–PDMS copolymer nanofibres for five thermomechanical cyclic tests.

Sample		Cycle 1	Cycle 2	Cycle 3	Cycle 4	Cycle 5
PCL_6_–PDMS_4_	R_f_ (%)	86.97	90.18	91.50	95.78	98.74
	R_r_ (%)	163.41	84.43	103.10	102.16	101.36
PCL_7_–PDMS_3_	R_f_ (%)	90.14	95.49	85.97	94.27	93.52
	R_r_ (%)	95.69	142.93	101.46	98.79	110.99
PCL_8_–PDMS_2_	R_f_ (%)	83.72	90.87	92.49	95.53	96.07
	R_r_ (%)	101.47	115.71	112.41	100.69	100.69
